# Low-Dose Lipopolysaccharide Pretreatment Suppresses Choroidal Neovascularization via IL-10 Induction

**DOI:** 10.1371/journal.pone.0039890

**Published:** 2012-07-03

**Authors:** Nagakazu Matsumura, Motohiro Kamei, Motokazu Tsujikawa, Mihoko Suzuki, Ping Xie, Kohji Nishida

**Affiliations:** Department of Ophthalmology, Osaka University Graduate School of Medicine, Osaka, Japan; Statens Serum Institute, Denmark

## Abstract

Recent studies have suggested that some kinds of microbial infection may have a crucial role in the development of many diseases such as autoimmune diseases and certain types of cancer. It has been reported that some chronic infections, such as *Chlamydia pneumoniae*, and immunological dysfunctions are associated with age-related macular degeneration (AMD), a leading cause of blindness. To evaluate the association between systemic low-level inflammation induced by infection and AMD pathogenesis, we investigated whether intraperitoneal injection of lipopolysaccharide (LPS) can modulate the development of laser-induced choroidal neovascularization (CNV), a key feature of AMD. Contrary to our expectations, the sizes of CNV in mice with LPS pretreatment were approximately 65% smaller than those of the control mice. After LPS pretreatment, serum IL-10 concentration and IL-10 gene expression in peritoneal macrophages and in the posterior part of the eye increased. Peritoneal injection of anti-IL10 antibody reduced CNV suppression by LPS pretreatment. Moreover, adoptive transfer of the resident peritoneal macrophages from LPS-treated mice into control littermates resulted in an approximately 26% reduction in the size of CNV compared with PBS-treated mice. We concluded that CNV formation was suppressed by low-dose LPS pretreatment via IL-10 production by macrophages.

## Introduction

Age-related macular degeneration (AMD) is a leading cause of legal blindness all over the world [1]. AMD is divided into dry and wet forms. Choroidal neovascularization (CNV), a characteristic feature of the wet type AMD, is currently a target for treatments, because it affects 90% of patients with severe visual loss due to AMD. Several types of treatments, including macular translocation surgery [2], [3], photodynamic therapy (PDT) [4], anti-VEGF therapy such as ranibizumab [5], and the combination therapy of PDT and ranibizumab [6] have recently been developed to treat CNV. However, the primary cause and the pathogenesis of AMD are not fully known, which results in suboptimal outcomes with the current therapies.

Increasing evidence suggests that immune-related processes may be involved in the pathogenesis of AMD [7], [8], [9], [10], [11], [12]. Considering the recent studies reporting that chronic and subclinical infections affect the function of the immune system and induce many diseases, such as autoimmune diseases, atherosclerosis and some kind of cancer [13], [14], [15], and that bacterial or viral infections like *Chlamydia pneumonia* and cytomegalovirus are associated with AMD incidence [16], [17], certain infections in a person with a predisposing condition, including genetic and environmental factors, may induce a dysfunction of the immune system and trigger the onset of AMD.

To investigate the relationship between systemic low-level bacterial infection and AMD pathogenesis, we examined the effects of pretreatment with lipopolysaccharide (LPS, endotoxin), a major component of Gram-negative bacteria walls, on an animal model of AMD. We demonstrate that low-dose LPS pretreatment suppresses laser-induced CNV via interleukin-10 (IL-10) secretion by peritoneal macrophages. These data suggest that macrophages stimulated by environmental agents like pathogens may play a protective role in the pathogenesis of AMD.

## Materials and Methods

### Animals

Male 8- to 10- week-old C57BL/6 mice were purchased from Japan Charles River Breeding Laboratories (Tokyo, Japan). AII mice were housed in pathogen-free conditions in the animal facility at Osaka University. The animals were cared for in accordance with the Association for Research in Vision and Ophthalmology (ARVO) Statement for the Use of Animals in Ophthalmic and Vision Research. The Institutional Animal Care and Use Committee of Osaka University specifically approved this study (#20–094-0). All animal experiments were carried out in accordance with a protocol approved by the committee. For all procedures, anesthesia was achieved by an intramuscular injection of 50 mg/kg ketamine (Sankyo, Co., Ltd., Tokyo, Japan) and 10 mg/kg xylazine (Rompun, Bayer AG, Leverkusen, Germany).

### Low-dose LPS Treatment

**Figure 1:**
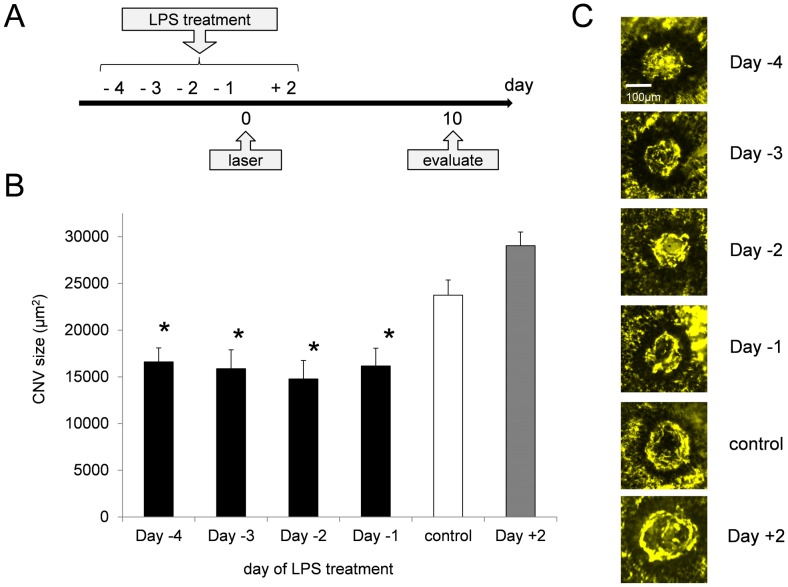
LPS pretreatment suppressed CNV formation. Peritoneal injection of low-dose LPS (20 µg) was performed at 4, 3, 2 or 1 days (respectively, Day -4, -3, -2 and -1) before laser irradiation (Day 0), or at 2 days after laser irradiation (Day +2), and the CNV size was evaluated at 10 days after laser treatment (Day 10) (A). In all groups of LPS-pretreated mice, the size of CNV was significantly smaller than that in control mice (B, C). The smallest CNV was shown in the mice given LPS pretreatment 2 days before laser treatment. The bars show means ± SEM. *n* = 6 mice/group, *P = 0.002 compared with control.

The mice were intraperitoneally injected with 20 µg of LPS (Escherichia coli 055:B5; Sigma–Aldrich, St. Louis, MO, USA, cat #L2880) in 200 µl phosphate-buffered saline (PBS). The LPS dose used in the current experiment was much lower than those used in previous studies to induce septic shock and endotoxin-induced uveitis (EIU) in mice [18], [19]; that is, those studies used approximately 100 to 200 µg, whereas we used only 20 µg in this experiment. There was no evidence of LPS toxicity in the animals as demonstrated by minimal weight loss and no behavioral change after injection. The intraperitoneal injections were performed at 4, 3, 2 or 1 days (respectively, Day -4, -3, -2 and -1) before laser irradiation (Day 0), or at 2 days after laser irradiation (Day +2) (Fig. 1A). The control mice received the same volume of PBS 2 days before laser irradiation.

### Laser-induced CNV Model

To induce CNV, the mice were anesthetized, and their pupils were dilated with a mixture of 0.5% phenylephrine and 0.5% tropicamide (Mydrin P, Santen Pharmaceutical, Osaka, Japan). Laser photocoagulation (532 nm, 150 mW, 100 ms, 75µm; Coherent 2000SE, Lumenis, Palo Alto, CA, USA) was performed as previously described [20]. Briefly, three laser burns were placed at the 3, 9, and 12 o’clock meridians centered on the optic nerve head and located 2 to 3 disk diameters from the optic nerve head in each eye, using a lit lamp delivery system and a coverslip as a contact lens. The morphologic end point of the laser injury was the appearance of a cavitation bubble, a sign of the disruption of Bruch’s membrane. Laser treatment was performed at 4, 3, 2 or 1 days after the LPS injection, or at 2 days before the LPS injection (Fig. 1A). Eyes showing subretinal or vitreous hemorrhage were excluded from the experiments.

**Figure 2:**
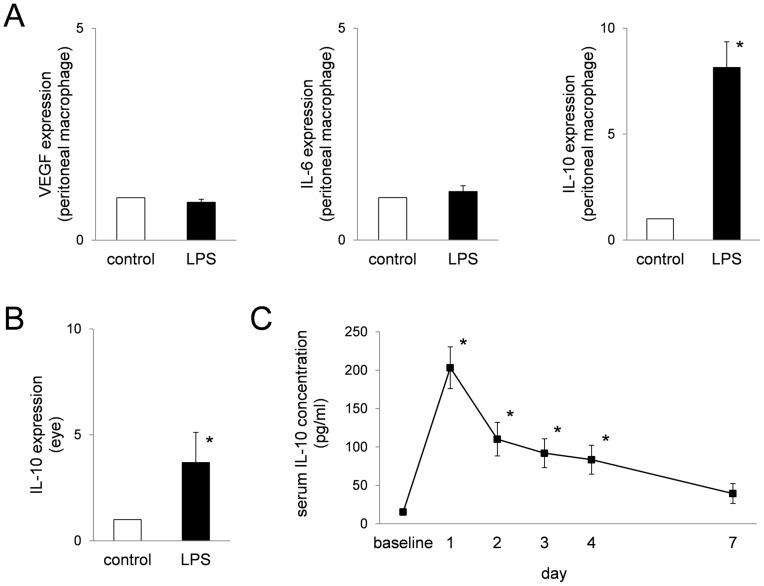
LPS treatment increased serum IL-10 concentration and IL-10 expression in peritoneal macrophages and in the eye. After peritoneal injection of low-dose LPS, IL-10 expression in the peritoneal macrophages (A) and in the posterior part of the eye (the retina, RPE and choroid) (B) increased, approximately 8-fold and 4-fold, respectively, two days after LPS injection. The bars show means ± SEM. *n* = 6 mice/group, *P<0.001 compared with control. Serum IL-10 concentration increased (C). *n* = 6, *P<0.001 compared with baseline. It reached a peak on day 1 and gradually decreased. A significant increase was shown for at least 4 days.

### Quantitative Analysis of CNV

Ten days after laser irradiation, the sizes of the CNV lesions were measured in retinal pigment epithelium (RPE)-choroid-sclera flat mounts as previously described [21]. Briefly, the mice were anesthetized and perfused through the left ventricle with 5 ml of fluorescein-labeled dextran (50 mg/ml, fluorescein isothiocyanatedextran; Sigma Aldrich) in PBS. The eyes were enucleated after euthanasia and fixed in 10% phosphate-buffered formalin for 3 hours. After the anterior segment and the vitreous were removed, the entire retina was carefully dissected from the eyecup. The remaining RPE-choroid-sclera complex was flat mounted on glass slides using Fluoromount Aqueous Mounting Medium (Diagnostic Biosystems, AC, USA) and coverslips after 4-7 relaxing radial incisions (average 5). Flat mounts were examined using a fluorescent microscope (BZ-9000, Keyence, Osaka, Japan) and images were captured. ImageJ software (developed by Wayne Rasband, National Institutes of Health, Bethesda, MD) was used to measure the area of CNV associated with each burn.

### Isolation and Purification of Peritoneal Macrophages

Peritoneal cells were collected by peritoneal lavage with ice-cold PBS, and incubated in a culture dish with DMEM containing 10% FBS (Invitrogen, Carlsbad, CA, USA) for 1 hour at 37°C in 5% humidified CO_2_ for purification of macrophages by adhesion. The nonadherent cells were removed by gentle washing three times with warm PBS. Because more than 95% of the adherent cells were F4/80-positive, we used them as peritoneal macrophages for quantitative real-time PCR and adoptive transfer experiments.

**Figure 3:**
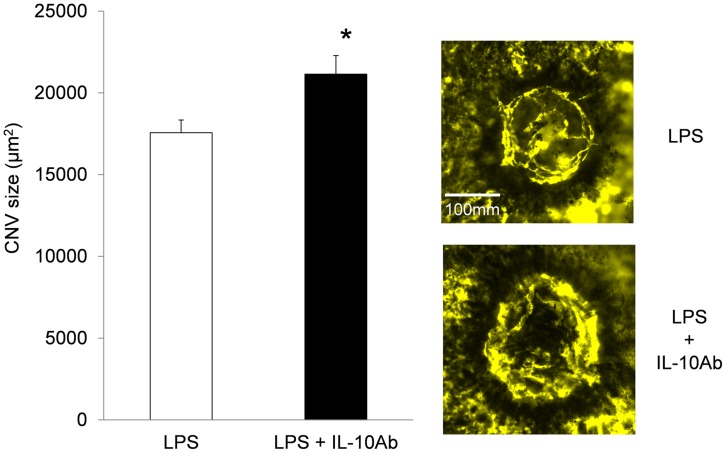
Anti-IL-10 antibody inhibited the CNV inhibitory effect of LPS pretreatment. Peritoneal injection of anti-IL-10 neutralizing antibody (IL-10Ab) inhibited the CNV inhibitory effect of LPS pretreatment in LPS-treated mice. The bars show means ± SEM. *n* = 6 mice/group, *P = 0.01.

### Quantitative Real-time PCR

Total RNA was extracted from these peritoneal macrophages and the posterior part of the eyeball (the retina, RPE and choroid) at 48 hours after laser treatment using Isogen (NIPPON GENE Co, Itd., Tokyo, Japan). Total RNA was reverse-transcribed into complementary DNA (cDNA) using Superscript III First Strand Synthesis System (Invitrogen, Carlsbad, CA, USA) and random hexamer primers, according to the manufacturer’s instructions. The gene expression was analyzed by a real-time quantitative PCR, using TaqMan Gene Expression Assays (Applied Biosystems, Foster City, CA, USA). Assay identification numbers used are as follows: *IL-10*, Mm00439614_m1; *IL-6*, Mm00446190_m1; *VEGF*, Mm00437304_m1; and *Actb*, Mm00607939_s1. To normalize for differences in efficiency of sample extraction or cDNA synthesis by reverse transcriptase, we used β-actin as a housekeeping gene. The ΔΔCT method was used for relative quantification.

### Serum IL-10 Measurement

Serum IL-10 levels were determined by the Quantikine Mouse IL-10 ELlSA kit (R&D Systems, Inc., Minneapolis, M N, USA) according to the manufacturer’s instructions before and at 1, 2, 3, 4 and 7 days after LPS injection.

**Figure 4:**
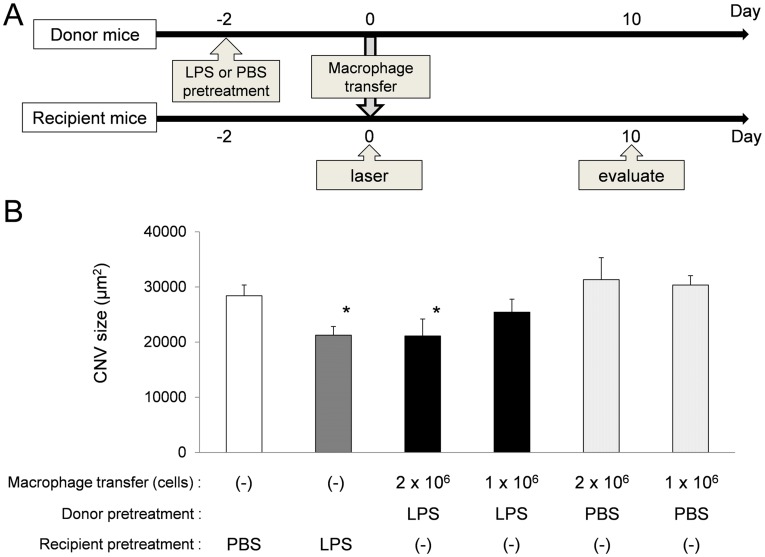
Adoptive transfer of LPS-treated peritoneal macrophages suppressed CNV formation as well as did LPS pretreatment. The donor mice were injected with LPS (20 µg/PBS 200 µl) or PBS (200 µl) at Day -2. At Day 0, laser treatment was performed on the recipient mice. After that, peritoneal macrophages were harvested from the donor mice and 2×10^6^ or 1×10^6^ macrophages were transferred into the peritoneal cavity of the recipient mice (A). For comparison, laser treatment was performed in PBS-pretreated mice and LPS-pretreated mice without adoptive transfer. In the LPS-pretreated mice and the recipient mice with 2×10^6^ macrophages from LPS-treated donor mice, CNV was significantly smaller than that in the control mice and the recipient mice with macrophages from PBS-pretreated donor mice (B). The bars show means ± SEM. *n* = 6 mice/group, *P = 0.003 compared with control.

### IL-10 Blockade with a Neutralizing Antibody

To confirm the contributory role of IL-10 in the development of CNV, we tried to block IL-10 with its neutralizing antibody. A total of 150 µg anti-IL-10 antibody (R&D Systems, Minneapolis, MN, USA, cat #AB-417-NA) in 1 µl PBS was intraperitoneally injected 2 days before laser treatment, simultaneously with or without LPS pretreatment. At 10 days after laser treatment, the size of CNV was evaluated.

### Adoptive Transfer of Peritoneal Macrophages

To investigate the involvement of peritoneal macrophages in CNV formation, adoptive transfer of peritoneal macrophages from LPS-treated mice to untreated mice was performed. Donor mice were pretreated with LPS (20 µg/PBS 200 µl) or PBS (200 µl) at Day -2. At Day 0, laser treatment was performed in the recipient mice and adoptive transfer of peritoneal macrophages was performed from the LPS- or PBS-treated donor mice to the recipient mice. A total of 2×10^6^ or 1×10^6^ peritoneal macrophages in 0.5ml PBS were injected into the peritoneal cavity of the recipient mice immediately after the laser treatment. For comparison, laser treatment was performed in PBS-pretreated mice and LPS-pretreated mice without adoptive transfer. The size of CNV lesions was measured at 10 days after laser irradiation.

### Statistical Analysis

Data are expressed as mean +- SEM. Statistical significance was analyzed by t-test for IL-10 blockage experiment, by Mann-Whitney Rank Sum Test for quantitative real-time PCR experiments and by Kruskal-Wallis One Way Analysis of Variance on Ranks for the other experiments. *P*<0.05 was considered statistically significant.

## Results

### Low-dose LPS Pretreatment Reduced the Size of CNV

To assess the effect of the low-dose LPS pretreatment on CNV formation, the mice were injected intraperitoneally with low-dose LPS (20 µg) before or after laser treatment to induce CNV formation (Fig. 1A). The areas of CNV in the mice with LPS pretreatment at 4, 3, 2 or 1 days (respectively, Day -4, -3, -2 and -1) before laser treatment were 16603, 15878, 14770 and 16171 µm^2^, respectively, significantly smaller than that of the control (23738 µm^2^; *P*<0.001, Kruskal-Wallis One Way Analysis of Variance on Ranks using Dunn’s method) (Fig. 1B-C). The area of CNV in the mice with LPS treatment at 2 days after laser treatment (Day +2) was 29030 µm^2^, which although larger than that of the control, was not statistically significant. These results show that low-dose LPS pretreatment does not increase CNV size, but instead suppresses CNV formation. The suppressive effect was significant in all of the pretreated mice, and the maximum effect was achieved in the mice given LPS at Day -2. The following experiments were, therefore, performed with LPS treatment at 2 days before the laser treatment.

### IL-10 is Involved in the CNV Inhibitory Effect by LPS

To reveal the mechanisms of CNV suppression by LPS, we investigated the involvement of some cytokines. VEGF, IL-6, IL-10. VEGF and IL-6 are angiogenic cytokines which have been reported to be associated with CNV formation [5], [22]. IL-10 is an anti-inflammatory and immunomodulatory cytokine mainly produced by immune cells, including macrophages [23], [24].

IL-10 gene expression increased by approximately 8-fold in peritoneal macrophages and 4-fold in the posterior part of the eye two days after LPS injection (Fig. 2A, B). The increases are statistically significant (*P*<0.001, Mann-Whitney Rank Sum Test). On the other hand, there was no significant change in both VEGF and IL-6 gene expression in peritoneal macrophages (Fig. 2A).

Serum IL-10 concentration increased after low-dose LPS administration and reached the maximum concentration one day after LPS administration (Fig. 2C). The IL-10 concentration then gradually decreased and returned the baseline level at 7 days after LPS administration, but was significantly elevated for at least 4 days (*P*<0.001, Kruskal-Wallis One Way Analysis of Variance on Ranks using Dunn’s Method).

We next determined whether IL-10 blockage inhibits the LPS-induced suppression of CNV. LPS-treated mice were injected intraperitoneally with anti-IL-10 antibody simultaneously with LPS treatment. The CNV size was significantly larger in the anti-IL-10 antibody-injected mice than in the mice without anti-IL-10 antibody injection (*P* = 0.01, t-test) (Fig. 3).

These results show that IL-10 is a major player in the CNV inhibitory effect of LPS.

### Adoptive Transfer of Peritoneal Macrophages from the LPS-treated Mice Suppressed CNV Formation

To confirm the involvement of peritoneal macrophages in CNV suppression in the LPS-treated mice, we performed adoptive transfer of peritoneal macrophages from LPS-treated mice to PBS-treated mice (Fig. 4A). The size of CNV in the recipient mice with 2×10^6^ macrophages transferred from LPS-treated mice was significantly smaller than that in the control mice by 26% (21132 µm^2^ and 28416 µm^2^, respectively) (*P* = 0.003, Kruskal-Wallis One Way Analysis of Variance on Ranks using Dunn’s method) (Fig. 4B). In the recipient mice with 1×10^6^ macrophages transferred from LPS-treated mice, the CNV was smaller than that in the control mice, but was not statistically significant. In contrast, the size of CNV was not suppressed in the mice with macrophages transferred from PBS-treated mice. These results strongly suggest that peritoneal macrophages activated by low-dose LPS play a pivotal role in CNV suppression by LPS pretreatment.

## Discussion

LPS, a major component of the cell membrane of Gram negative bacteria, induces a strong immune response in normal animals. LPS is perceived by the infected host as a primary pathogen-associated molecular pattern (PAMP) via the Toll-like receptor 4 (TLR4), followed by the production of proinflammatory cytokines such as TNFα, IL-1β, and IL-6. Therefore, we at first expected that LPS pretreatment would increase CNV formation because bacterial infections, such as *Chlamydia pneumoniae* infection, were reported to be associated with an increased incidence of AMD [25]. However, contrary to our expectations, our data demonstrated that low-dose LPS pretreatment suppresses the formation of experimental CNV.

To uncover the details of the mechanisms of the LPS suppressive effect, we focused on IL-10, which was upregulated in peritoneal macrophages after LPS pretreatment. LPS not only initiates acute inflammatory responses but also induces anti-inflammatory cytokine production by immune cells. IL-10 is an anti-inflammatory and immunomodulatory cytokine produced by a number of cell types in humans, including monocytes, macrophages, B-lymphocytes, and Th-2 cells [24]. Peritoneal macrophages have been shown to produce IL-10 upon LPS stimulation [23]. In this study, we confirmed that serum, intraperitoneal and intraocular IL-10 levels increased after LPS administration. We also confirmed IL-10 involvement by blocking its action with intraperitoneal injections of anti-IL-10 antibody; that is, CNV size was not reduced in the eyes of mice injected with both LPS and anti-IL-10 antibody. These results show that IL-10 produced by the peritoneal macrophages is a major player in the CNV inhibitory effect of LPS pretreatment.

However, the role of IL-10 in CNV formation remains controversial. Apte et al. reported that IL-10 knockout mice have significantly reduced CNV compared to wild type, suggesting that IL-10 inhibited recruitment of macrophages to CNV area and promoted CNV formation [26]. On the other hand, Hasegawa et al. suggested that IL-10 had anti-angiogenic properties on CNV formation [27]. Our results showed that IL-10 was a suppressive agent on CNV formation. The effect of IL-10 on CNV may vary according to the amount of IL-10 and the inflammatory microenvironment.There are some limitations in this study, such as differences between laser-induced CNV in mice and human CNV. Macrophage activation pivotally participates in the pathophysiology of chronic inflammatory diseases including AMD, while macrophages in laser-induced CNV accumulate as an acute inflammation. It is unknown whether infiltrating macrophages have a protective or facilitatory role in the pathogenesis of AMD [28]. It has been shown that macrophages are plastic and that the microenvironment can influence the polarization of the macrophages toward the proinflammatory phenotype or the anti-inflammatory phenotype [29]. In our model, it is possible that LPS pretreatment changes the character of macrophages into the anti-angiogenic variety. Our experiments were performed in an animal model with an acute inflammatory response. It is necessary to investigate further with a chronic inflammation model.

Our data suggest that microbial infection may have an inhibitory effect on AMD, although some kinds of infection give a predisposing cause for other diseases [13], [14], [15]. It has been reported that prolonged exposure to endotoxin during the first year of life protects from asthma and atopic dermatitis. This is known as the hygiene hypothesis [30]. Our data propose a new concept about the pathogenesis of AMD; environmental exposure to some kinds of pathogens, including LPS from some bacteria, can induce subclinical changes in the immune system and reduce excess reactions against additive stimuli, such as the oxidative stress induced by blue light and high-fat food. So a history of microbial infection can play a protective role in the pathogenesis of AMD.

In summary, our results suggest that low-dose LPS pretreatment has a suppressive effect on CNV formation via IL-10 secreted by macrophages stimulated by this pretreatment. If we can control macrophage plasticity and create the same conditions in the eye using drugs, it will be a potentially useful therapy for AMD in the future.
